# COVID-19 and Hypertensive Disorders of Pregnancy (HDP): A Portuguese Multicentric Retrospective Cohort Study of HDP in SARS-CoV-2 Infected Patients

**DOI:** 10.7759/cureus.36483

**Published:** 2023-03-21

**Authors:** Ana Rita Mira, António De Pinho, Mónica Calado-Araújo, Catarina Ferreira, Daniela David, Margarida Enes, Carolina Vaz-de-Macedo

**Affiliations:** 1 Obstetrics and Gynaecology, Hospital Garcia de Orta, Almada, PRT; 2 Obstetrics and Gynaecology, Centro Hospitalar Tâmega e Sousa, Penafiel , PRT; 3 Obstetrics and Gynaecology, Unidade Local de Saúde de Matosinhos, Porto, PRT; 4 Obstetrics and Gynaecology, Hospital Professor Doutor Fernando Fonseca, Lisboa, PRT; 5 Obstetrics and Gynecology, Centro Hospitalar e Universitário de Coimbra, Coimbra, PRT

**Keywords:** gestational hypertension, pre-eclampsia, pregnancy-associated hypertensive disorders, covid-19, sars-cov-2

## Abstract

Background: An increased incidence of hypertensive disorders of pregnancy (HDP) has been reported among pregnant women infected by the severe acute respiratory syndrome coronavirus-2 (SARS-CoV-2), the pathogen behind coronavirus disease-19 (COVID-19). Although it is primarily a respiratory infection, the extra-pulmonary manifestations of COVID-19 mimic those found in preeclampsia (PE). Moreover, the two conditions share common risk factors and pathological mechanisms, hindering the ability to understand the interaction between them. Current literature on this topic is controversial and as there is an overlap of clinical and laboratory findings, HDP can be an overreported outcome in pregnant women with COVID-19. The aim of our study is to assess whether there is an association between maternal SARS-CoV-2 infection and HDP.

Methods: We designed a multicenter retrospective cohort study with data collected from five maternity hospitals in Almada, Porto, Lisboa, Penafiel and Coimbra, Portugal, between March 2020 and March 2021. We obtained a sample of 789 pregnant women who were followed up or delivered their babies in one of the participating centers. Each pregnant woman who tested positive for SARS-CoV-2 on a real-time polymerase chain reaction test -- exposure group (n= 263), was paired with two negative pregnant women (1:2), who received the same antenatal care and had similar gestational age and parity -- control group (n=526). Data were collected on maternal characteristics, medical history, obstetric outcomes, and delivery.

Outcomes: The primary outcome of our study is to assess the incidence of HDP in pregnant women infected and not infected by SARS-CoV-2. The secondary outcomes of our study are to assess the incidence of HDP across all COVID-19 severity subgroups and to assess whether SARS-CoV-2 infection in pregnancy modified the odds of a set of risk factors developing HDP.

Results: There was a slightly increased, but not statistically significant, incidence of PE (relative risk, RR, 1.33; 95% confidence interval, CI 0.68-2.57) in the SARS-CoV-2 positive group. There was no statistically significant association between having COVID-19 in pregnancy and developing PE/eclampsia/ hemolysis, elevated liver enzymes, and low platelets, HELLP syndrome [X2(1) = 0.732; p = 0.392] as well as developing gestational hypertension (GH) [X2(1) = 0.039; p = 1]. There was no statistically significant association [X^2^(2) = 0.402; p = 0.875), [X^2^(2) = 1.529; p = 0.435] between COVID-19 severity and incidence of HDP. The SARS-CoV-2 infection did not modify the odds of each maternal risk factor causing HDP.

Conclusion: Our study did not demonstrate an association between maternal COVID-19 and HDP. We did not observe a significantly increased incidence of HDP in pregnant women infected by SARS-CoV-2. As current literature is controversial on this topic, clinicians should be aware that HDP is a possible complication of maternal SARS-CoV-2 infection and further research studies urge to better assess the association between COVID-19 in pregnancy and HDP.

## Introduction

Hypertensive disorders of pregnancy (HDP) are a spectrum of pregnancy disorders secondary to blood pressure elevation, including gestational hypertension (GH), preeclampsia (PE), eclampsia and hemolysis, elevated liver enzymes, and low platelets (HELLP) syndrome [1]. PE affects 2%-5% of pregnant women and is one of the leading causes of maternal and perinatal morbidity and mortality [2]. An increased incidence of HDP has been reported among pregnant women infected by the novel severe acute respiratory syndrome coronavirus-2 (SARS-CoV-2) [3-4], the pathogen behind coronavirus disease 2019 (COVID-19) [5]. Although COVID-19 is primarily a respiratory infection, it can have several extra-pulmonary manifestations, such as acute kidney lesions, liver injuries, thrombocytopenia, coagulation abnormalities, and hypertension [4-6]. These lesions are secondary to renin-angiotensin axis dysregulation, viral dissemination, and exaggerated immune response mediated by inflammatory cytokines that cause endothelial dysfunction and multi-organ damage [6-7]. Since COVID-19 extra-pulmonary manifestations mimic clinical and laboratory features of PE, its differential diagnosis can be challenging [8]. In addition to their clinical resemblance, COVID-19 and HDP share common risk factors such as age, body mass index, and hyperglycemia in pregnancy, which can confound their association [9]. Moreover, they seem to share a common pathological mechanism of abnormal maternal circulation related to endothelial dysfunction [10] leading to placental perfusion compromise [11]. Altogether these observations hinder our ability to understand the interaction between COVID-19 and HDP and we hypothesize that HDP may be overreported among SARS-CoV-2-infected pregnant women. The primary aim of this study is to assess whether there is an association between maternal COVID-19 and HDP by evaluating and comparing the incidence of HDP in pregnant women infected and not infected by SARS-CoV-2, in a Portuguese cohort. 

## Materials and methods

Study design

We developed a multicenter cohort retrospective study to assess the association between COVID-19 in pregnancy and HDP, namely GH and PE/eclampsia/HELLP syndrome. Data were collected from five maternity hospitals in Almada, Porto, Lisboa, Penafiel and Coimbra, Portugal, during the period between March 2020 and March 2021. Pregnant women who were followed up or delivered their babies during this period in one of the participant centers were enrolled in the study. 

Sample size calculation was obtained by taking as a reference The INTERCOVID Multinational Cohort Study [3]. We calculated a sample size of 700 participants -- 230 SARS-CoV-2 positive cases and 470 SARS-CoV-2 negative cases -- assuming statistical significance at p < 0.05 and 80% study power.

Data were collected retrospectively by examination of electronic health records. All women with a diagnosis of COVID-19 at any stage of their pregnancy and labor, defined by a positive real-time polymerase chain reaction (RT-PCR) test for SARS-CoV-2, were included in the study as the exposed group. Two pregnant women with a negative RT-PCR test for SARS-CoV-2, who received the same antenatal care and had similar gestational age and parity were enrolled for each positive case (1:2 ratio), forming the control group. Control group selection obeyed some principles: 1) inpatient clinic: selection of the first two PCR-SARS-CoV-2 negative pregnant women who were admitted on the same day as the index case, who met pairing criteria in the order presented by the informatic system. Gestational age pairing was according to the following groups -- < 20 weeks, 20-24 weeks, 25-27 weeks, 28-31 weeks, 32-33 weeks, 34-36 weeks, and > 37 weeks; 2) outpatient clinic: selection of the first two PCR-SARS-CoV-2 negative pregnant women who had an appointment on the same day as the index case, when she had her COVID-19 diagnosis and who met pairing criteria. Gestational age pairing was according to pregnancy trimester. When any of the above criteria were not met, the two PCR-SARS-CoV-2 negative pregnant women that were temporally closer to the index case were selected.

Live and stillborn pregnancies were included in addition to those with congenital anomalies. All multiple gestation pregnancies were excluded as they account for different risks of obstetric outcomes than singleton pregnancies. Data were collected on maternal characteristics, medical history, obstetric outcomes, and delivery.

Outcomes

The primary outcome was to assess whether there is an increased incidence of HDP, specifically GH and PE/eclampsia/HELLP syndrome in pregnant women infected by SARS-CoV-2 compared to women not infected by SARS-CoV-2. The two secondary outcomes were to evaluate and compare the incidence of HDP according to COVID-19 severity and to assess whether SARS-CoV-2 infection in pregnancy modified the odds of certain risk factors contributing to the development of HDP. 

Preeclampsia was defined by systolic blood pressure (SBP) ≥ 140 mmHg and/or diastolic blood pressure (DBP) ≥ 90 mmHg on at least two occasions measured 4 h apart in previously normotensive women with an onset at or after 20 weeks gestation and one of the following new-onset conditions: proteinuria, maternal organ, and/or uteroplacental dysfunction. Eclampsia was defined as the occurrence of convulsions or comas unrelated to cerebral conditions in a woman with symptoms and signs of PE. HELLP syndrome was defined as evidence of hemolysis, with lactate dehydrogenase (LDH) elevated to 600 IU/L or more, elevated liver enzymes more than twice the upper limit of normal, and platelet count less than 100 x 109/L. GH was defined as SBP ≥ 140 mmHg and/or DBP ≥ 90 mmHg on at least two occasions measured 4 h apart after 20 weeks of gestation in a previously normotensive woman and absence of proteinuria. COVID-19 severity was obtained according to the United States National Institutes of Health (NIH) guidelines in five categories: asymptomatic or presymptomatic infection, mild illness, moderate illness, severe illness, and critical illness [12].

Data management and statistical analysis

Collected data were inserted into a database and managed according to the general data protection regulations (GDPR). We used IBM SPSS version 26 (IBM Corp., Armonk, NY) for the statistical analysis. Continuous variables were expressed as mean and standard deviation. Categorical variables were expressed in absolute and relative frequency. Some categorical variables were expressed as dichotomic outcomes (e.g., PCR SARS-CoV-2), whilst others were expressed in categories (e.g., COVID-19 symptoms). We first compared the baseline demographic characteristics and medical histories of the two study groups -- the PCR SARS-CoV-2 positive and negative women -- using a student’s t-test for numeric variables and a Chi-square test for categorical variables. We secondly assessed the incidence of HDP -- GH and PE/eclampsia/HELLP syndrome -- in the SARS-CoV-2 positive and SARS-CoV-2 negative groups. We further evaluated the incidence of HDP in SARS-CoV-2 positive subgroups organized according to COVID-19 severity -- asymptomatic, mild, and moderate/severe/critical. For our primary outcome, we assessed whether there was an association between having COVID-19 in pregnancy and developing PE/eclampsia/HELLP syndrome using the Chi-square test, and GH using Fisher’s Exact test. For our secondary outcomes, we used Fisher’s Exact test to assess the association between COVID-19 severity and HDP development. Also for our secondary outcomes, we performed univariable and multivariable logistic regression models to analyze whether SARS-CoV-2 infection modified the odds of a set of risk factors, known to be associated with both COVID-19 and HDP, developing GH and PE/eclampsia/HELLP. Results were expressed as relative risk (RR) or odds ratio (OR) and 95% confidence interval (CI). We set statistical significance at p < 0.05.

Ethics approval

Ethics approval was obtained from all participating medical institutions -- ethical approval committee number 87/2021. The study was conducted following the principles of the Declaration of Helsinki.

## Results

We enrolled a total of 789 pregnant women. Of these, 33.3% (n = 263) were diagnosed with COVID-19 during pregnancy and 66.7% (n = 526) constituted the control group. All women were non-vaccinated for SARS-CoV-2. The contribution of each center to the total study population ranged from 1.9% (n = 15) -- Centro Hospitalar Universitário de Coimbra to 69.6% (n = 549) -- Hospital Garcia de Orta. Other centers contributed to the study data as follows: 6.8% (n= 54) -- Centro Hospitalar Tâmega e Sousa; 7.6% (n = 60) -- Hospital Professor Doutor Fernando Fonseca; 14.1% (n = 111) -- Unidade Local de Saúde de Matosinhos. The groups of women with and without COVID-19 had similar demographic characteristics and medical histories (Table 1). However, women in the COVID-19 group were younger with a mean maternal age of 29 years vs 31 years [t(787) = 3.275; p = 0.001], had less smoking habits - 7.6% (n = 20) vs 15.6% (n = 82) [X2(1) = 10.917; p = 0.0019] and had lower nulliparity rates -- 41.4%, (n = 109) vs 50.8%, (n = 267) [X2(1) = 6.1; p = 0.014] than women in the COVID-19 negative group.

**Table 1:** Demographic characteristics and clinical history of women with and without COVID-19 diagnosis. COVID-19, coronavirus disease 2019; n, number

	Overall (n=789)	Women without COVID-19 diagnosis (n=526)	Women with COVID-19 diagnosis (n=263)
Demographic characteristics			
Maternal age (years)			
	30,9 ± 6,1	31,4 ± 6,0	29,9 ± 6,0
Body Mass Index (kg/m^2^)			
	26,3 ± 5,4	26,2 ± 5,4	26,3 ± 5,3
Underweight	16 (2,0%)	11 (2,1%)	5 (1,9%)
Healthy	257 (32,6%)	173 (32,9%)	84 (31,9%)
Overweight	190 (24,1%)	128 (24,3%)	62 (23,6%)
Obesity	135 (17,1%)	92 (17,5%)	43 (16,3%)
Omiss	191 (24,2%)	122 (23,2%)	69 (26,2%)
Smoking habits			
No	540 (68,4%)	343 (65,2%)	197 (74,9%)
Yes	102 (2,9%)	82 (15,6%)	20 (7,6%)
Omiss	147 (18,6%)	101 (19,2%)	46 (17,5%)
Parity			
Nulliparous	376 (47,7%)	267 (50,8%)	109 (41,4%)
Multiparous	413 (52,3%)	259 (49,2%)	154 (58,6%)
Clinical history			
History of hypertensive disorders of pregnancy			
No	766 (97,1%)	512 (97,3%)	254 (96,6%)
Yes	23 (2,9%)	14 (2,7%)	9 (3,4%)
History of hyperglicemia in pregnancy			
No	674 (85,4%)	447 (85,0%)	227 (86,3%)
Yes	115 (14,6%)	79 (15,0%)	36 (13,7%)
History of chronic hypertension			
No	756 (95,8%)	505 (96,0%)	251 (95,4%)
Yes	33 (4,2%)	21 (4,0%)	12 (4,6%)
History of chronic kidney disease			
No	785 (99,5%)	523 (99,4%)	262 (99,6%)
Yes	4 (0,5%)	3 (0,6%)	1 (0,4%)
History of autoimmune disease			
No	787 (99,7%)	524 (99,6%)	263 (100,0%)
Yes	2 (0,3%)	2 (0,4%)	0 (0,0%)

The median gestational age for COVID-19 diagnosis was 34 weeks (interquartile range, 31-39). Of the 263 women with COVID-19, 65.4% (n = 172) were asymptomatic, 27.0% (n = 71) had a mild illness, and 4.2% (n = 11) had moderate/severe/critical illness.

**Figure 1:**
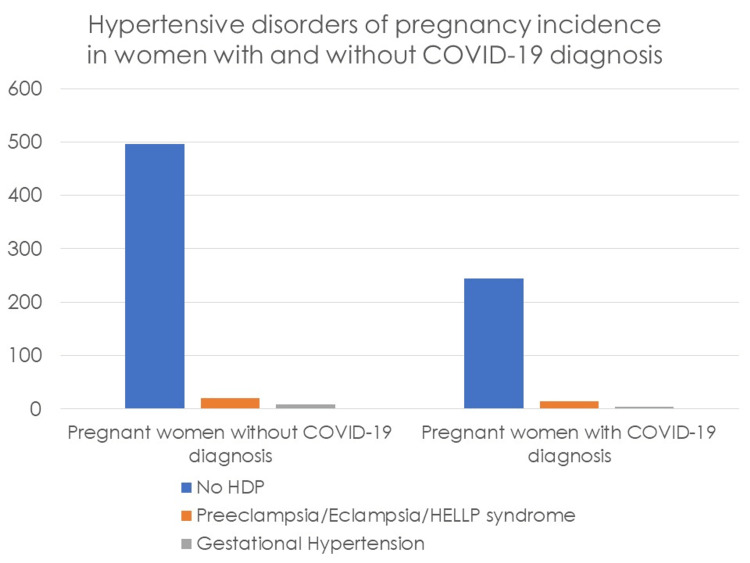
Incidence of hypertensive disorders of pregnancy in pregnant women with and without COVID-19 diagnosis. COVID-19, coronavirus disease 2019; HDP, hypertensive disorders of pregnancy; HELLP syndrome, hemolysis, elevated liver enzymes and low platelets syndrome

Overall, 1.6% (n = 13) women had GH and 4.4% (n = 35) had PE/eclampsia/HELLP syndrome. Of the women who had PE/eclampsia/HELLP syndrome, 5.3% (14/263) were in the SARS-CoV-2 positive group, whereas 4.0% (21/526) were in the SARS-CoV-2 negative group (RR, 1.33; 95% CI 0.68-2.57) (Figure 1). Of the women who had GH, 1.7% (4/263) were in the SARS-CoV-2 positive group and 1.5% (9/526) were in the SARS-CoV-2 negative group (RR, 0.88; 95% CI 0.27-2.85) (Figure 1).

In our primary outcome analysis -- the incidence of HDP in pregnant women infected and not infected by SARS-CoV-2 -- we verified the absence of a statistically significant association between having COVID-19 in pregnancy and developing PE/eclampsia/HELLP syndrome [X2(1) = 0.732; p = 0.392] as well as developing GH [X2(1) = 0.039; p = 1].

**Figure 2:**
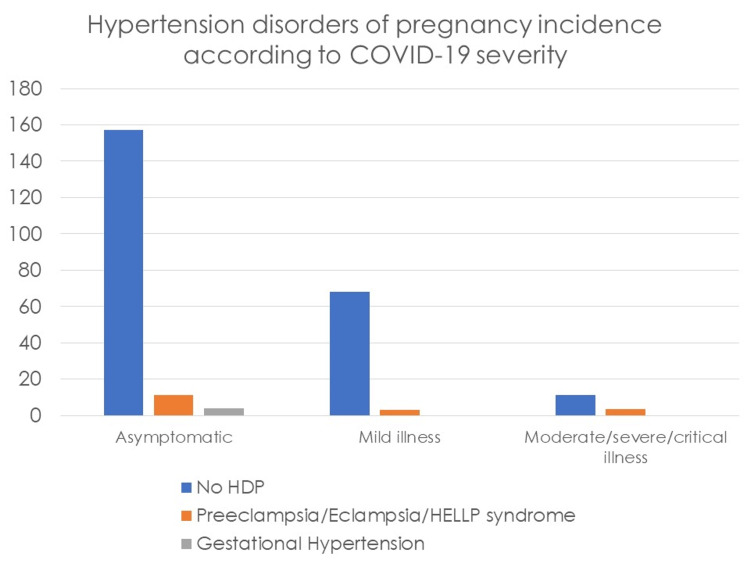
Distribution of hypertensive disorders of pregnancy among COVID-19 severity groups. COVID-19, coronavirus disease 2019; HDP, hypertensive disorders of pregnancy; HELLP syndrome, hemolysis, elevated liver enzymes and low platelets syndrome

In our secondary outcomes analysis -- the incidence of HDP among COVID-19 severity subgroups -- we concluded that there was no statistically significant association in all spectrums of COVID-19 severity and development of PE/eclampsia/HELLP syndrome [X2(2) = 0.402; p = 0.875) and GH [X2(2) = 1.529; p = 0.435] (Figure 2).

Additionally, in our secondary outcomes analysis, we explored whether SARS-CoV-2 infection modified the odds of several risk factors developing HDP. First, we used a univariable logistic regression model to determine the odds of developing PE/eclampsia/HELLP syndrome and GH for each risk factor. Body mass index (OR = 1,067; CI 95% = 1.002-1.135), HDP antecedents (OR = 8.97; CI 95% = 3.293-24.429), and chronic hypertension (OR = 7; CI 95% = 2.801-17.494) were the three factors that independently had a statistically significant association with PE/eclampsia/HELLP syndrome. Maternal age (OR = 1.107; CI 95% = 1.006-1.218), HDP antecedents (OR = 6.537; CI 95% = 1.363-31.351), hyperglycemia in pregnancy antecedents (OR = 3.784; CI 95% = 1.216-11.778), and smoking habits (OR = 3.633; CI 95% = 1.007-13.109) had an independent statistically significant association with the development of GH. Second, we designed a multivariable logistic regression model that combined the variables of maternal SARS-CoV-2 infection and HDP risk factors. After adding SARS-CoV-2 infection to the model, the odds of developing PE/eclampsia/HELLP syndrome and GH did not change significantly (Table 2).

**Table 2:** OR of each maternal risk factor for the development of hypertensive disorders of pregnancy after adjustment for SARS-CoV-2 infection in pregnancy. SARS-CoV-2, severe acute respiratory syndrome coronavirus-2; HELLP syndrome, hemolysis, elevated liver enzymes and low platelets syndrome; OR, odds ratio; CI, confidence interval; BMI, body mass index

Multivariable logistic regression adjusting each risk factor for SARS-CoV-2 infection outcome: preeclampsia/eclampsia/HELLP syndrome		
	OR	CI
Maternal age	1,023	0,967-1,082
BMI	1,067	1,002-1,135
Parity	1,536	0,772-3,057
Smoking habits	0,6	0,177-2,033
History of hypertensive disorders of pregnancy	8,857	3,246-24,166
History of chronic hypertension	6,959	2,781-17,415
History of hyperglycemia in pregnancy	1,508	0,642-3,540
History of autoimmune disease	0	-
History of chronic kidney disease	7,601	0,766-75,424
Multivariable logistic regression adjusting each risk factor for SARS-CoV-2 infection outcome: gestational hypertension		
	OR	CI
Maternal age	1,108	1,006-1,220
BMI	1,067	0,969-1,174
Parity	1,277	0,423-3,849
Smoking habits	3,641	0,987-13,425
History of hypertensive disorders of pregnancy	6,599	1,373-31,714
History of chronic hypertension	0	-
History of hyperglicemia in pregnancy	3,776	1,213-11,756
History of autoimmune disease	0	-
History of chronic kidney disease	0	-

Because we found some differences in maternal characteristics between our SARS-CoV-2 infected and our control groups, we further adjusted our analysis for the factors: parity, maternal age, and smoking habits. The relative risk for PE did not change significantly despite the adjustment - nulliparous (RR 1.31; 95% CI 0.54-3.21) vs multiparous (RR 1.47; 95% CI 0.54-3.98); non-smokers (RR 1.19; 95% CI 0.56-2.52) vs smokers (RR 2.04; 95% CI 0.19-21.27); maternal age < 35 (RR 1.39; 95% CI 0.62-3.12) vs maternal age ≥ 35 (RR 1.26; 95% CI 0.39-4.06) -- so as the relative risk of GH -- nulliparous (RR 0.4; 95% CI 0.04-3.35) vs multiparous (RR 1.68; 95% CI 0.34-8.19); maternal age <35 (RR 0.36; 95% CI 0.04-3.08) vs maternal age ≥ 35 (RR 1.89; 95% CI 0.43-8.26).

## Discussion

Main findings

This is the first multicentric cohort study developed in Portugal to assess specifically the association between maternal SARS-CoV-2 infection and HDP. Despite observing an increased incidence of HDP in pregnant women infected by SARS-CoV-2 compared to our control group, this difference was not statistically significant, meaning that our study failed to demonstrate an association between maternal SARS-CoV-2 infection and HDP. 

In our secondary outcomes analysis, we did not observe any statistically significant difference in the incidence of HDP between all the COVID-19 severity groups, when assuming that symptom occurrence is indicative of a worse disease and worse pregnancy outcomes. Additionally, we observed that COVID-19 did not modify the odds of certain maternal risk factors -- maternal age, parity, body mass index, HDP antecedents, chronic hypertension, smoking habits, and hyperglycemia in pregnancy -- developing HDP.

Interpretation and hypothesized explanation

Preeclampsia is a pregnancy condition, whose physiopathology is not fully understood, but it is known to involve endothelial dysregulation, imbalance of angiogenic factors, coagulopathy, and placental injury. Its main clinical feature is hypertension after 20 weeks of gestation which is accompanied by multiple organ damage such as renal injury, hepatic injury, and neurologic symptoms.

In pregnancy, maternal immune response to SARS-CoV-2 infection triggers a systemic inflammatory cascade marked by cytokine dysregulation, endothelial dysfunction, and coagulopathy [13], causing multiple organ injuries which ensue clinical and laboratory features similar to those seen in PE [8].

Multiple studies in the scientific literature report an increased incidence of PE and GH among SARS-CoV-2-infected pregnant patients [4, 14-18]. The common theme of abnormal maternal circulation [11] shared between PE and the immune response to SARS-CoV-2 can be a possible explanation for the increased incidence of HDP.

However, it is still not currently known whether this association is causal due to the lack of specific biomarkers to distinguish the two conditions. A group of scholars [8] described PE-like syndrome as the set of clinical and laboratory features secondary to SARS-CoV-2 infection that resemble those of PE. Due to the similar presentation and lack of specific biomarkers, it is possible that PE has been overreported among pregnant women infected by SARS-CoV-2. 

As per our main findings, we failed to demonstrate the primary and secondary outcomes of our study, therefore, we failed to demonstrate an association between maternal SARS-CoV-2 infection and HDP. Our results are in line with those of other international studies published in the scientific literature, reporting no increase in the incidence of HDP among SARS-CoV-2 positive pregnant women [19-22].

The disparity of results found in the scientific literature depicts the need for further studies with methodologies capable of distinguishing PE cases from those that are a result of maternal immune response to SARS-CoV-2.

Clinical implications

COVID-19 in pregnancy is known to be associated with greater maternal, perinatal, and neonatal morbidity and mortality [3]. Our data provide further information about COVID-19 and HDP in a sample of 789 pregnant women in Portugal.

Taking into account the results of our study and the conflicting evidence of literature on this topic, clinicians should be aware that HDP is a possible complication of SARS-CoV-2 infection in pregnancy, despite their association being still not well established. A high level of suspicion should be raised in women with a diagnosis of COVID-19 and closer pregnancy monitoring should be ensured.

Furthermore, it is essential to boast clinical signs that are more specific to the maternal immune response to SARS-CoV-2 infection and distinctive PE symptoms in order to improve the ability to differentiate the two conditions and assess more precisely the incidence of HDP.

Simultaneously, the focus of researchers should be directed to establishing specific biochemical and placental histopathological markers of maternal SARS-CoV-2 infection to better assess its effect in pregnancy.

Scholars should conduct further international cohort studies on HDP and COVID-19, using control groups, to improve the quality of evidence of this topic. 

Strengths and limitations

This large multicentric cohort study was designed specifically to assess the incidence of HDP. Unlike many published studies that looked at maternal morbidity indexes and composed outcomes of maternal SARS-CoV-2 infection, our study improves the quality of evidence in this topic’s literature due to its objectivity.

Our large sample size was obtained from a conservative sample size calculation with a 2:1 ratio of negative: positive cases which strengthened the power of the study. We used a rigorous methodology for sample collection to minimize selection bias and improve the validity of our results.

Despite our pairing criteria, we verified statistically significant differences in some maternal characteristics between groups, such as maternal age, nulliparity, and smoking habits. Maternal age and parity are PE risk factors that could have contributed to the overall number of HDPs accounted for in the control group. However, we overruled this effect by adjusting our analysis for these factors and verified that our results did not change.

Since a large proportion of our sample was collected from Hospital Garcia de Orta, we acknowledge that we must be cautious in interpreting our findings. Additionally, we acknowledge that we cannot extrapolate our results to the general population. 

## Conclusions

Our study did not demonstrate an association between maternal SARS-CoV-2 infection and HDP. We did not observe a significantly increased incidence of HDP in pregnant women infected by SARS-CoV-2 compared to the non-infected group. This observation was consistent across all spectra of COVID-19 severity. Further international cohort studies urge to understand whether there is an association between COVID-19 and HDP as the current literature is controversial on this topic. Clinicians should be aware of this possible pregnancy outcome and monitor patients accordingly. Future research should focus on establishing clinical, biochemical, and placental histopathological markers specific to SARS-CoV-2 infection in pregnancy to better assess the incidence of HDP in pregnant patients with COVID-19. 

## References

[1] Brown MA, Magee LA, Kenny LC (2018). The hypertensive disorders of pregnancy: ISSHP classification, diagnosis &amp; management recommendations for international practice. Pregn Hypertens.

[2] Poon LC, Shennan A, Hyett JA (2019). The International Federation of Gynecology and Obstetrics (FIGO) initiative on pre-eclampsia: a pragmatic guide for first-trimester screening and prevention. Int J Gynaecol Obstet.

[3] Villar J, Ariff S, Gunier RB (2021). Maternal and neonatal morbidity and mortality among pregnant women with and without COVID-19 infection: the INTERCOVID multinational cohort study. JAMA Pediatr.

[4] Papageorghiou AT, Deruelle P, Gunier RB (2021). Preeclampsia and COVID-19: results from the INTERCOVID prospective longitudinal study. Am J Obstet Gynecol.

[5] Huang C, Wang Y, Li X (2020). Clinical features of patients infected with 2019 novel coronavirus in Wuhan, China. Lancet.

[6] Gupta A, Madhavan MV, Sehgal K (2020). Extrapulmonary manifestations of COVID-19. Nat Med.

[7] Bohn MK, Hall A, Sepiashvili L, Jung B, Steele S, Adeli K (2020). Pathophysiology of COVID-19: mechanisms underlying disease severity and progression. Physiology (Bethesda).

[8] Mendoza M, Garcia-Ruiz I, Maiz N (2020). Pre-eclampsia-like syndrome induced by severe COVID-19: a prospective observational study. BJOG.

[9] Rosenbloom JI, Raghuraman N, Carter EB, Kelly JC (2021). Coronavirus disease 2019 infection and hypertensive disorders of pregnancy. Am J Obstet Gynecol.

[10] Palomo M, Youssef L, Ramos A (2022). Differences and similarities in endothelial and angiogenic profiles of preeclampsia and COVID-19 in pregnancy. Am J Obstet Gynecol.

[11] Shanes ED, Mithal LB, Otero S, Azad HA, Miller ES, Goldstein JA (2020). Placental pathology in COVID-19. Am J Clin Pathol.

[12] (2022). United States of America National Institutes of Health - Clinical Spectrum of SARS-CoV-2 Infection. https://www.covid19treatmentguidelines.nih.gov/overview/clinical-spectrum/.

[13] Gurol-Urganci I, Jardine JE, Carroll F (2021). Maternal and perinatal outcomes of pregnant women with SARS-CoV-2 infection at the time of birth in England: national cohort study. Am J Obstet Gynecol.

[14] Simon E, Gouyon JB, Cottenet J, Bechraoui-Quantin S, Rozenberg P, Mariet AS, Quantin C (2022). Impact of SARS-CoV-2 infection on risk of prematurity, birthweight and obstetric complications: a multivariate analysis from a nationwide, population-based retrospective cohort study. BJOG.

[15] Litman EA, Yin Y, Nelson SJ, Capbarat E, Kerchner D, Ahmadzia HK (2022). Adverse perinatal outcomes in a large United States birth cohort during the COVID-19 pandemic. Am J Obstet Gynecol MFM.

[16] Epelboin S, Labrosse J, De Mouzon J (2021). Obstetrical outcomes and maternal morbidities associated with COVID-19 in pregnant women in France: a national retrospective cohort study. PLoS Med.

[17] Metz TD, Clifton RG, Hughes BL (2021). Disease severity and perinatal outcomes of pregnant patients with coronavirus disease 2019 (COVID-19). Obstet Gynecol.

[18] Snelgrove JW, Simpson AN, Sutradhar R, Everett K, Liu N, Baxter NN (2022). Preeclampsia and severe maternal morbidity during the COVID-19 pandemic: a population-based cohort study in Ontario, Canada. J Obstet Gynaecol Can.

[19] Guida JP, Cecatti JG, Souza RT (2022). Preeclampsia among women with COVID-19 during pregnancy and its impact on maternal and perinatal outcomes: results from a national multicenter study on COVID in Brazil, the REBRACO initiative. Pregn Hypertens.

[20] Daclin C, Carbonnel M, Rossignol M (2022). Impact of COVID-19 infection in pregnancy and neonates: a case control study. J Gynecol Obstet Hum Reprod.

[21] Crovetto F, Crispi F, Llurba E (2021). Impact of severe acute respiratory syndrome coronavirus 2 infection on pregnancy outcomes: a population-based study. Clin Infect Dis.

[22] Bernard I, Limonta D, Mahal LK, Hobman TC (2020). Endothelium infection and dysregulation by SARS-CoV-2: evidence and caveats in COVID-19. Viruses.

